# Genomes from Verteba cave suggest diversity within the Trypillians in Ukraine

**DOI:** 10.1038/s41598-022-11117-8

**Published:** 2022-05-04

**Authors:** Pere Gelabert, Ryan W. Schmidt, Daniel M. Fernandes, Jordan K. Karsten, Thomas K. Harper, Gwyn D. Madden, Sarah H. Ledogar, Mykhailo Sokhatsky, Hiroki Oota, Douglas J. Kennett, Ron Pinhasi

**Affiliations:** 1 Department of Evolutionary Anthropology, University of Vienna, Vienna, Austria; 2 Human Evolution and Archaeological Sciences, University of Vienna, Vienna, Austria; 3 University of Porto, CIBIO-InBIO, Rua Padre Armando Quintas, nº 7, 4485-661 Vairão, Portugal; 4 School of Archaeology & Earth Institute, University College, Dublin, Belfield, Dublin 4, Ireland; 5 CIAS, Department of Life Sciences, University of Coimbra, Coimbra, Portugal; 6 Department of Anthropology, Global Religions, and Cultures, University of Wisconsin-Oshkosh, 800 Algoma Blvd, Oshkosh, WI 54901 USA; 7 Institute for European and Mediterranean Archaeology, State University of New York at Buffalo, Buffalo, NY 14260 USA; 8 Department of Anthropology, Grand Valley State University, 1 Campus Dr., Allendale, MI 49401 USA; 9 Department of Archaeology, Classics, and History, University of New England, Armidale, NSW 2351 Australia; 10 Borschiv Regional Museum of Local Lore, Borschiv, Ternopil Oblast Ukraine; 11 Department of Biological Sciences, Graduate School of Science, University of Tokyo, 7-3-1 Hongo, Bunkyo-ku, Tokyo, 113-0033 Japan; 12 Department of Anthropology, University of California, Santa Barbara, CA 93106 USA

**Keywords:** Anthropology, Archaeology, Population genetics

## Abstract

The transition to agriculture occurred relatively late in Eastern Europe, leading researchers to debate whether it was a gradual, interactive process or a colonisation event. In the forest and forest-steppe regions of Ukraine, farming appeared during the fifth millennium BCE, associated with the Cucuteni-Trypillia cultural complex (CTCC, ~ 5000–3000 BCE). Across Europe, the Neolithisation process was highly variable across space and over time. Here, we investigate the population dynamics of early agriculturalists from the eastern forest-steppe region based on the analyses of 20 ancient genomes from the site of Verteba Cave (3935–825 cal BCE). Results reveal that the CTCC individuals’ ancestry is related to both western hunter-gatherers and Near Eastern farmers, has no local ancestry associated with Ukrainian Neolithic hunter-gatherers and has steppe ancestry. An Early Bronze Age individual has an ancestry profile related to the Yamnaya expansions but with 20% of ancestry related to the other Trypillian individuals, which suggests admixture between the Trypillians and the incoming populations carrying steppe-related ancestry. A Late Bronze Age individual dated to 980–825 cal BCE has a genetic profile indicating affinity to Beaker-related populations, detected close to 1000 years after the end of the Bell Beaker phenomenon during the third millennium BCE.

## Introduction

The Neolithisation process in Europe resulted in dramatic technological and cultural shifts, which included novel subsistence practices^1^. There are two major groups of models that explain the Neolithisation process: demic diffusion models describe Neolithisation as a colonisation process by farmers which is propelled by exponential population growth characteristic of the Neolithic, whereas acculturation models outline the process as one in which at least some of the transition entails indigenous hunting-foraging groups that adopt farming following periods of variable length during which they interact with neighbouring exogenous farmers^2^. Across most of Europe, the Neolithic transition was genetically defined by a profound population replacement, consistent with the demic diffusion of peoples from Anatolia^3–5^_._ The Anatolian farmers reached the Balkans and other regions of Southeast Europe in the seventh millennium BCE^6,7^ and subsequently spread further via the Mediterranean and later through the Danube, substantially replacing indigenous Mesolithic European populations^8,9^.

In contrast to Central Europe^10,11^, areas of Eastern Europe including Ukraine, Moldova, Western Russia, and Romania did not adopt agriculture until the Late Neolithic (~ 4500 BCE), although various sedentary and semi-sedentary hunter-gatherers Mesolithic groups in these regions began using pottery as early as 8500 BCE^12,13^.

The Cucuteni-Trypillia cultural complex (CTCC) is a grouping of several interrelated Middle Neolithic/Eneolithic archaeological cultures in parts of Ukraine, Moldova, and Romania^14,15^. This complex stretches from the Transylvanian Alps to the Dnieper River and is named for the type-sites of Cucuteni in Iași County, Romania and Trypillia (also known as Tripolye, in Russian) in Kyiv oblast, Ukraine. The Cucuteni and Trypillia cultures have common roots in the Precucuteni culture; the earliest CTCC sites are found in the piedmont of the Carpathian Mountains and the earliest radiocarbon dates (from the Precucuteni 2 period) date to around 4800 BCE^16,17^. The CTCC originated from the interaction of several Danubian Neolithic groups, with evidence for similarities in house construction, ceramic styles, and lithic artifact production^10,16,18,19^.

Following the origin of this cultural complex in the Carpathian piedmont, the CTCC eventually occupied a territory covering much of the modern territories of Ukraine, Moldova, and Romania. The first diagnostically Early Trypillia (Trypillia A) sites diverged from the Precucuteni culture ~ 4500 BCE in the Dniester River valley. Later population movements, occurring from the middle period (Trypillia BI) onward, saw the Trypillia culture expand to Volhynia in the west and the Dnieper River in the east. This territorial expansion is believed to have resulted primarily from demographic increases associated with a successful agropastoral subsistence strategy, and the search for new arable land for cultivation^18^. However, some population growth may have been the product of Trypillian populations incorporating indigenous hunting and gathering (HG) groups, such as members of the Bug-Dneister culture. Another mode of population increase could have been the acculturation of refugees following the collapse of the Neolithic in Romania, Hungary, and Bulgaria. During the middle-to-late periods of the Trypillia culture (Trypillia BII to CI; 4100–3400 BCE), some CTCC groups established extremely large settlements in Central Ukraine, often referred to as “giant-settlements'' or “megasites,” which attained sizes of 100–320 ha^20^. Rapid demographic growth within the CTCC around the turn of the fourth millennium BCE necessitated the exploitation of new territories, precipitating migrations to previously peripheral areas^21^.

Hypotheses for the rise of the megasites in particular are varied; it has been suggested that they may have been a defensive response to threats posed by steppe pastoralists or competing sub-groups within the CTCC^22,23^, or that they simply represent ephemeral episodes of population agglomeration due to large-scale migration from the Dniester region^24^.

**Figure 1:**
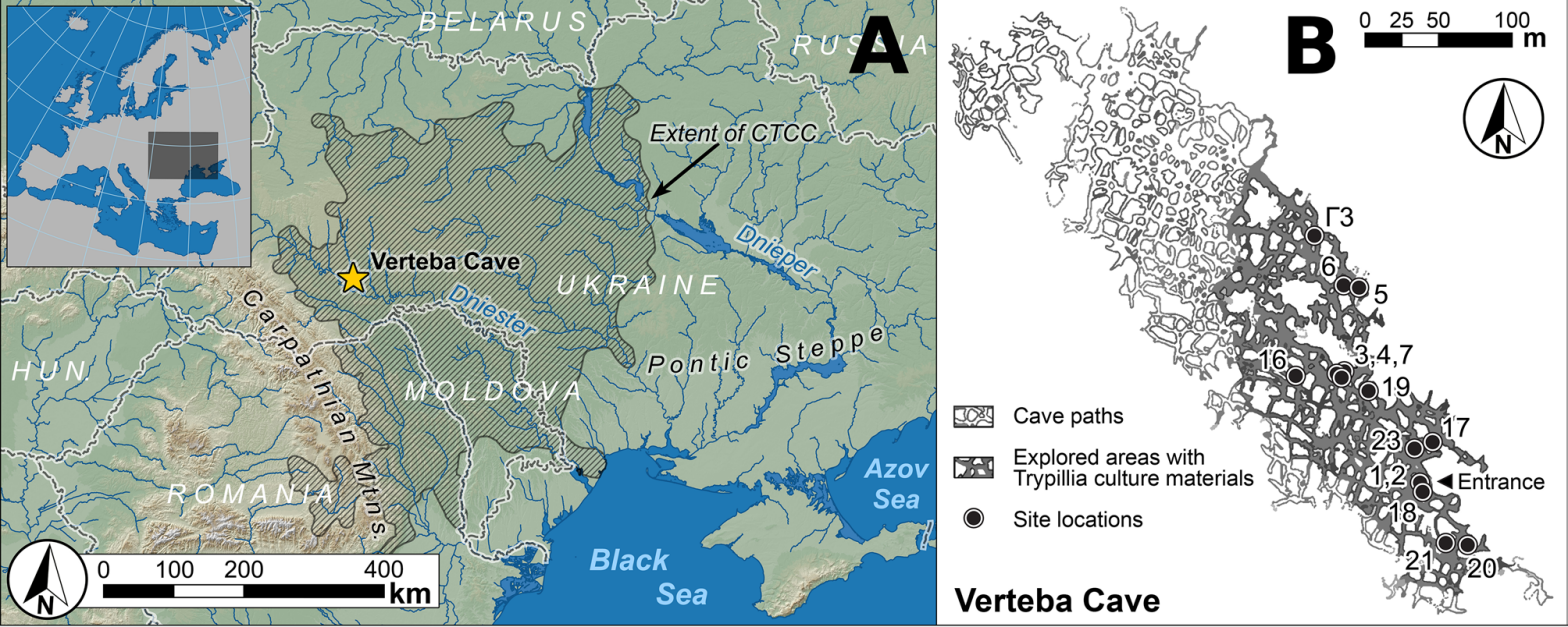
(**A**) Location of Verteba cave in Ternopil Oblast, Ukraine, plotted against the overall distribution of CTCC sites. (**B**) Map of sites within Verteba cave; individuals included in this publication were found at sites 7, 17 and 20, Adapted from Ledogar et al.^31^.

Even though Trypillian populations established a high density of settlements in Western and Central Ukraine^25^, very few burials have been located. Only a handful of cemeteries dating to the Late Trypillia period were excavated during the 1960s and 1970s, such as Chapaievka in Ukraine^26^ and Vykhvatyntsi in Moldova^27^. While these sites give some glimpse into Trypillian mortuary behavior, they are limited in their temporal scope and have not been subjected to modern laboratory analyses. To better understand the origins, connections, and diversity of the CTCC, we collected human remains from three chambers at the site of Verteba Cave (VC) in Ternopil oblast, Ukraine, one of the few sites that contain human remains associated with the CTCC (Fig. 1). Accelerator mass spectrometer (AMS) ^14^C dates of human and faunal remains place the Trypillian occupation of VC to between 3950 and 3520 cal BCE (2σ)^28,29^. On the basis of the ceramic assemblages present in the cave and a sample of lower-resolution liquid scintillation ^14^C dates, it is probable that occupation continued for some time into the Late Trypillia (CII) period and Early Bronze Age transition^30^. More recently, AMS radiocarbon dating has also identified deposits at different locations throughout the cave dating to the Mesolithic (7950–7490 cal BCE [2σ]), Bronze Age, Iron Age and Medieval period^31^. Skeletal assemblages were taken from three separate chambers (Site 7, Site 17, and Site 20) of the cave (Table S2, Fig. 1). Each of these chambers contain CTCC material culture; however, burials in the cave are secondary in nature and disturbance through human activity during antiquity and bioturbation complicate reconstruction of the cave’s use and chronology. Most individuals in this study come from Site 7, which has been extensively documented through ceramic analysis and radiocarbon dating^31,32^, with peak occupation dated to periods CI and early CII of the Trypillia periodization (~ 3900–3350 BCE). Interpretations regarding the use of the cave are varied, including its use as a temporary shelter, ritual site or mortuary location^29,33,34^. There is additional evidence to support the idea that the burials in the cave, which are largely commingled and secondary in nature, are representative of victims of warfare or sacrifice^35^_._

The paleogenetics of the Trypillian population is limited to the analysis of uniparental markers (mtDNA) and genome-wide analysis of 8 individuals. Mitochondrial haplogroups typical of ancient Eurasian farming groups (H, HV, T, K, J) have been observed for these individuals scattered throughout the cave^30,32,36^. Schmidt et al. found within a single chamber evidence for Haplogroup W, which has been observed for steppe populations associated with the Corded Ware and Unetice cultures of the middle Volga region^8^. Genome-wide analyses of CTCC individuals have shown ancestral components predominately of Early Neolithic farming groups (estimated to be 60–80%), confirming that the early farmers who settled Western and Central Ukraine were largely derived from the same source population as the farmers of Anatolia and Western Europe^13,37^. The remaining 20 percent of their ancestry is less certain. Mathieson et al. (2018) found that this ancestral component was a mix of western (WHG) and eastern (EHG) hunter-gatherers (HG) found in HG groups inhabiting the region during the Neolithic. Immel et al. recovered genome-wide data from four individuals buried at two separate sites in northern Moldova that date to 3500–3100 cal BCE (during the Late Trypillia period), five centuries younger than the radiocarbon dates from Verteba cave, and found a larger degree of steppe-related ancestry (albeit in varying proportions among the sampled individuals). This observation may be explained by the gradual assimilation of local Mesolithic and Neolithic HG groups into the Trypillian population, at least for groups who settled in Moldova.

The settlement systems of the Trypillia culture interacted with both Central European and the steppe populations. Archaeological evidence for steppe interaction is found in shell-tempered pottery, which are similar to steppe-style wares^35,38^. Some of these look nearly identical to pottery found at steppe sites, while others combine shell tempering with CTCC decorative motifs^35^. Symbolic objects influenced by—or directly imported from—steppe communities, such as stone mace heads, are found at some Middle-to-Late Trypillia sites^35^, and exchanges of pottery are evident as early as Trypillia BII^39^. There was undoubtedly some degree of interaction between Trypillian populations and the Dnieper-Donets culture, while any synchrony between the Trypillia culture and the following Yamnaya horizon was likely very brief. Regardless, however, some Trypillian populations were likely in permanent contact with steppe populations^40^. Interestingly, after around 3400 BCE, the Trypillian mega-sites were largely abandoned. The cause of this abandonment has been widely debated, one hypothesis is an increase in conflict due to the westward expansion of steppe populations. Such a hypothesis may find corroboration in the frequent evidence of violent death discovered in Verteba cave^23,35^.

Here, we recovered genome-wide sequence data from 20 individuals buried in VC, eight of which are directly dated by AMS ^14^C to the interval of 3790 to 825 cal BCE (Table S1). We use this data to specifically test several questions: (1) is there evidence for admixture with local HGs, as has been suggested by Rascovan et al.^29^; (2) using our expanded dataset with higher coverage than in Mathieson et al.^13^, can we clarify the Neolithic ancestral component of the Trypillian population, i.e., can we show that they are more similar to early farmers from Anatolia, the LBK (Linearbandkeramik), or elsewhere; (3) since the CTCC individuals lived in close proximity to steppe populations, is there evidence for genetic admixture with the Yamnaya or earlier steppe populations; and (4) do later Bronze Age populations that settled in the region share genetic affinities with the CTCC group from Verteba Cave?

## Results

DNA was extracted from 20 petrous bones. Eight of the samples were directly radiocarbon dated and we determined that six (VERT-035, VERT-106, VERT-031, VERT-100, VERT-104 and VERT-015) date to 3790–3535 cal BCE (2σ; Late Eneolithic), one individual (VERT-113) from Site 7 dates to 1960–1770 cal BCE (Middle Bronze Age; MBA) and one from Site 17 (VERT-114) dates to 980–825 cal BCE (Late Bronze Age; LBA) (Table S2). Endogenous content of the sequenced samples ranged from 59 to 82% and yielded genomic coverages between 0.2X and 2.2X (Table S2). Aligned reads were authenticated using dedicated ancient DNA pipelines (Supplementary methods). All analyzed sequences showed the typical pattern of aDNA: an excess of C>T transition in the 5’ end and G>A transitions in the 3’ end, consistent with the age of the samples; additionally, no signs of contamination were found in the sequences^41^. Further details of the contamination assessment is provided in Supplementary information. We were able to assign molecular sex to all the individuals, from which 8 are female and 12 are male (Table S2). In all cases the molecular sex is concordant with morphological sex. No familiar relationships have been identified in the analyzed data.

### Uniparental markers

The analyzed individuals that are generally thought to come from the Eneolithic Period have maternal haplogroups T2b, H, HV, K1, N1, J1, U5 and T2c (Table S2). The MBA sample shows haplogroup HV, typical from several Neolithic cultures such as the ALPC^5^ as well as European Bronze Age individuals^42,43^. The LBA individual shows haplogroup T2, also associated with multiple BA individuals and cultures^43–45^. These haplogroups are typically found in European Neolithic and Bronze Age populations^5,13,43^. Male individuals exhibit Y-chromosome haplogroups G, I and C, which have also been previously reported in Neolithic and Bronze Age populations of Europe^13^. Both the mtDNA and Y-chr haplogroups of all individuals are fully concordant with the previously reported data. (Table S2).

### Population genetics

**Figure 2:**
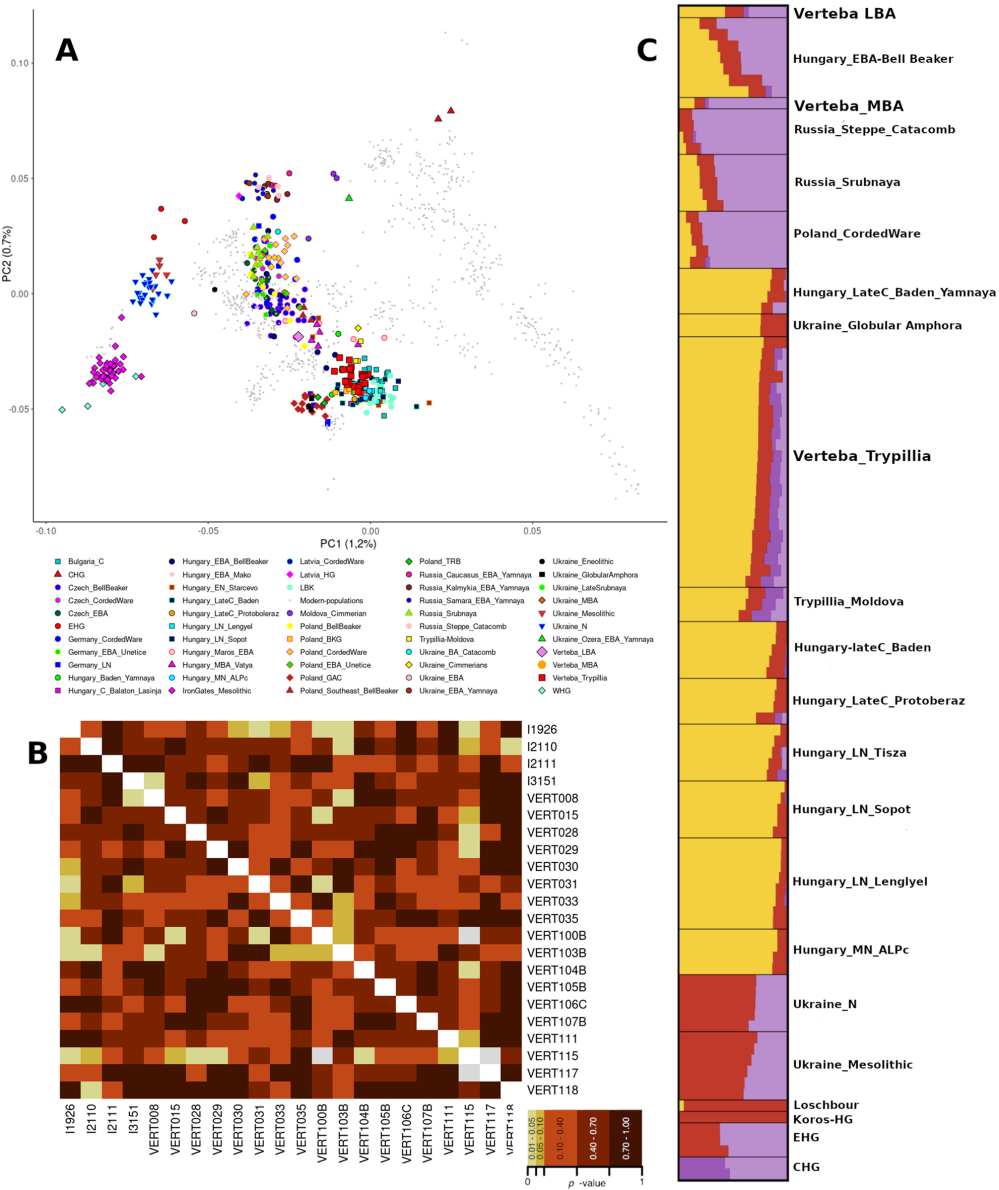
Population genetics: (**A**) PCA built with modern European populations in which Neolithic and Bronze Age populations of Eastern Europe have been projected. It is observed that the Verteba_Trypillia individuals are located within the European Neolithic populations genetic diversity while the Verteba_MBA and Verteba_LBA individuals are located close to other Bronze Age individuals suggesting genetic similarity. (**B**) ADMIXTURE analysis of the most representative populations included in the analysis (K = 4). The different colors represent the source ancestries of the studied individuals: Yellow represents Anatolia_N related ancestry, Red represents WHG related ancestry and the purple colors represent Steppe related ancestries, each individual is represented by the proportions of these ancestries. (**C**) Heatmap built with 1 × 1 qpWave results of the Verteba_Trypillia individuals, where no individuals show a sign to be clustered in a different population suggesting that these individuals belong to the same genetic population.

To place VC individuals within present-day and archaeological Eurasian populations, we used a principal component analysis (PCA)^46^ built with 729 modern individuals from Europe and the Mediterranean Basin^3^. Together with the 20 Verteba genomes, 478 additional ancient genomes (Table S3) were projected onto the PCA. 18 out of the 20 VC individuals are placed closely to Neolithic and Eneolithic European populations such as LBK, Central European Middle and Late Neolithic samples and the Moldova Trypillian individuals^37^ (Fig. 2A). The PCA also evidenced the extreme similarity between the 18 newly reported Trypillians and the other four Trypillians from Verteba Cave previously sequenced^13^, therefore all these 22 individuals were labeled together as Verteba_Trypillia and further analyzed together. The two Bronze Age individuals are clear outliers. Individual VERT-114 falls within the Bell-Beaker diversity and appears to have a position close to the Czech, Hungarian and Polish Bell-Beaker groups. Individual VERT-113 appears close to European Corded-Ware and Srubnaya populations, showing a strong affinity to steppe samples. We have then explored the presence of structure in the Trypillian population (only using the 22 samples from the main cluster) using qpWave. The results have shown the absence of population structure, therefore all samples have been analyzed together, as no individual showed statistically significant pairwise differences to the rest using a threshold of 0.05 (Fig. 2B).

We then explored the genetic diversity of the VC individuals using ADMIXTURE^47^. The 22 individuals (labeled ‘Verteba_Trypillia’) in the PCA that showed affinity with Eneolithic samples are mostly defined by the ancestral component dominant in Anatolia-Neolithic individuals, which suggests a strong relationship with European Neolithic populations, similar to previous studies^13,37^. However, these samples also show the presence of EHG, CHG, and WHG components as described in Mathiesson et al.^13^, with the exception of one individual (I3151), who seems to be absent of any EHG/CHG ancestry. Individual VERT-114 (LBA) shows a predominant Anatolia Neolithic component and a great presence of an EHG component. The MBA individual (VERT-113) exhibits a high degree of similarity with Corded Ware and Yamnaya steppe populations (Fig. 2C, Fig. S1).

**Figure 3:**
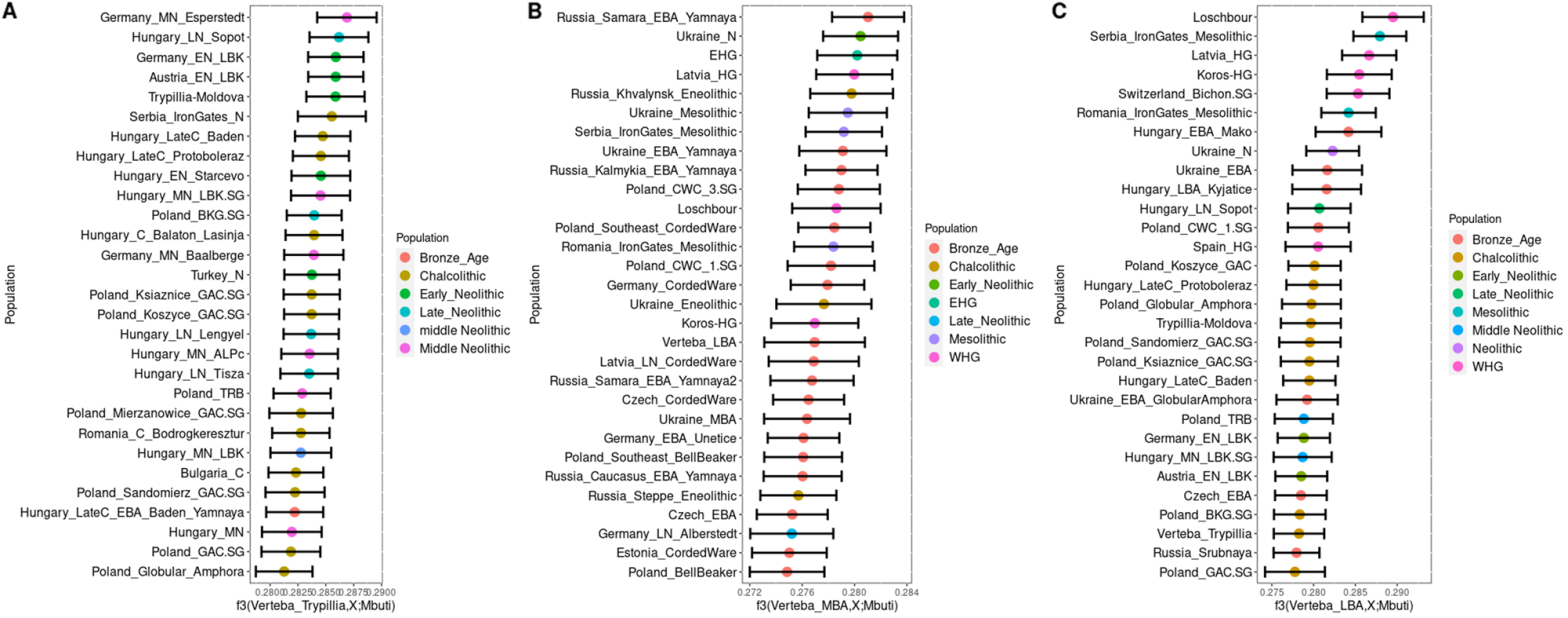
Outgroup-*f*_*3*_ statistics, the samples have been clustered between: Verteba individuals, individual VERT-114 (Verteba_LBA) and individual VERT-113 (Verteba_MBA). We have plotted the 20 populations with the highest values.

Next, we investigated the genetic affinities of the VC individuals using *f*-statistics. We used *f*_*3*_-outgroup statistics to quantify the amount of shared genetic drift of Verteba_Trypillia, VERT-114, and VERT-113, tested against other ancient European populations. Overall, the Verteba_Trypillia individuals share more derived SNPs with Neolithic European populations (Fig. 3). Individual VERT-114 shows a high level of derived SNPs with HG populations as well as with Late Neolithic and Bronze Age populations. In turn, individual VERT-113 shares derived SNPs with HG populations and some Steppe-related populations such as Central European Corded-Ware.

**Figure 4:**
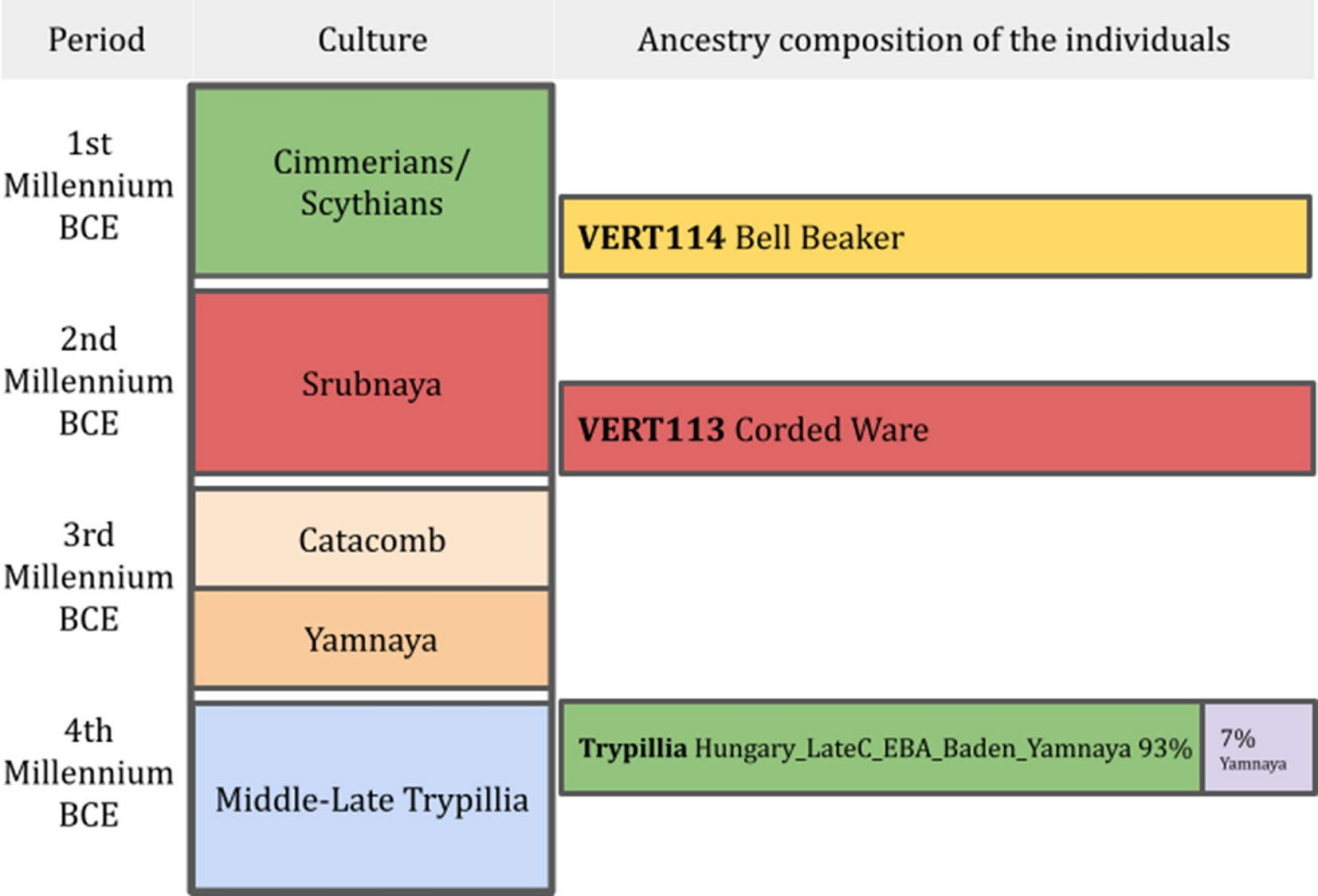
Ancestry and culture summary: Chronology of the different cultures that settled in Ukraine between the 4th and the 1st Millenium BCE, on the right the ancestry components of the analyzed individuals from Verteba cave according to the qpAdm analyses. The colors on the period column represent the different historical periods in the west of Ukraine. The Ancestry composition column colors graphically show the results of qpAdm. Green corresponds to Hungary_LateC_EBA_Baden_Yamnaya, Violet to Yamnaya, Red to Corded_Ware and Yellow to Bell_beaker.

We ran *f*_*4*_ statistics and qpAdm^46^ to quantify ancestry components as well as to estimate the direction of gene flow (Table S4, Fig. S2). We ran several tests to understand the genetic composition of Verteba_Trypillia (excluding the outliers) and the possible sources of genetic admixture for this population. We first ran qpAdm using populations chronologically close to the CTCC individuals. five models worked (Table S5), with the simplest ones involving about 93% of Hungary_LateC_EBA_Baden_Yamnaya ancestry plus 7% from Yamnaya-related populations, evidencing the connection between Trypillians and steppe populations as Hungary_LateC_EBA_Baden_Yamnaya also has Steppe ancestry (Fig. 4). We then tested possible connections with specific steppe-related populations using *f*_*4*_ statistical test in the form *f*_*4*_(Mbuti,Verteba_Trypillia;Russia_Samara_EBA_Yamnaya,Ukraine_EBA_Yamnaya) = − 0.000525, Z score = − 1.843, which does not statistically connects the Trypillians with the Ukrainian or Russian Yamnaya populations.

We then explored the same individuals, but this time using populations representing different basal ancestries that might have contributed to the CTCC genetic pool (EHG, CHG, Anatolia_N, WHG and Ukranie_N) (Table S6). Only one model worked, which involved 40% of Anatolia Neolithic-related ancestry, 20% of WHG and 40% of CHG. These results were also observed in Mathiesson et al. with very similar proportions, but were not feasible in the Moldovan Trypillians from Immel et al. To investigate the possible sources of the HG component of the 22 Verteba_Trypillia individuals we ran statistics in the form of: *f*_*4*_(Mbuti,Verteba_Trypillia;Ukraine_N,Serbia_IronGates_Mesolithic) = 0.001603, Z score = 12.173, *f*_*4*_(Mbuti, Verteba_Trypillia;Ukraine_N,Loschbour) = 0.001730, Z score = 6.380, *f*_*4*_(Mbuti, Verteba_Trypillia;Ukraine_N,Koros-HG) = 0.002037, Z score = 7.573, *f*_*4*_(Mbuti, Verteba_Trypillia;Koros-HG,Serbia_IronGates_Mesolithic) = − 0.00047, Z score = − 1.879. Showing that Koros-HG is the WHG source with the highest genetic affinity to Ukraine Trypillians. When compared to Central European Eneolithic populations, the Eneolithic Verteba individuals do not seem to share a statistically significant affinity with the CTCC population of Moldova, as shown in the form of *f*_*4*_(Mbuti, Verteba_Trypillia;Hungary_LateC_EBA_Baden_Yamnaya, Trypillia_Moldova):,-0.000304 Z score = − 0.936. qpAdm also indicates that Ukraine_N and WHG are two likely sources of HG-related ancestry to Verteba_Trypillia, in addition to Hungary_LateC_EBA_Baden_Yamnaya (Table S5). The statistic *f*_*4*_(Mbuti, Verteba_Trypillia; Ukraine_N, WHG) shows a clear tendency towards WHG: 0.001192, Z score = 6.26, suggesting very little presence of ancestry from the local Hunther_Gatherers in the Verteba individuals.

To detect different individual ancestry compositions, we ran qpAdm tests individually on the 22 Verteba_Trypillian individuals showing that most of them can be modeled with a single source using: Trypillia_Moldova, Hungary_LateC_EBA_Baden_Yamnaya or Hungary_LN_Tisza, indicating a clear affinity for Late_Neolithic populations with steppe ancestry (Table S7) as all these populations show the presence of steppe component. Surprisingly, only four of the Verteba_Trypillia individuals can be modeled using Trypillia-Moldova as a single source. To investigate if there are statistically significant differences between the possible sources for these individuals we ran the tests *f*_*4*_(Mbuti, VERTXXX; Hungary_LateC_EBA_Baden_Yamnaya, Trypillia-Moldova) individually (Table S8). The results show that only one individual (VERT-035) is statistically more related to Hungary_LateC_EBA_Baden_Yamnaya than to Trypillian-Moldova, pointing towards the existence of some variability within the Trypillians in Ukraine. As we did for the general population, we also performed the qpAdm analyses using distal sources (EHG, CHG, Anatolia_N, WHG and Ukranie_N). The results show that most of the individuals can be modeled using Anatolia_N plus a ~ 20% of WHG/Ukranie_N. When the *f*_*4*_(Mbuti, VERTXXX; Ukraine_N, WHG) is tested individually the statistics show no differences (Table S9), however the affinity to WHG at a population level is clear revealing the importance of having big sample sizes to perform f-statistics based assessments (Table S4).

VERT-113, dated to the MBA, shows a clear signal of steppe-related ancestry, and is the only individual in the dataset that shows a strong influx of this ancestry: *f*_*4*_(Mbuti, Verteba_MBA; Russia_Samara_EBA_Yamnaya, LBK): -0.00398 Z score = − 7.848. The same test with individual VERT-114 was not statistically significant (Z = 1.382). Relevantly, we observe a major affinity to Russia_Yamnaya over Ukraine_Yamnaya using *f*_*4*_ statistics (Table S5). Furthermore, this is the only individual that shows a major affinity to Ukranian_N over WHG as the source of HG-related ancestry, as shown by the statistic *f*_4_(Mbuti, VERT-113; Ukraine_N, WHG): − 0.001276, Z score (Z = − 4.202). The distal models of qpAdm using basal ancestries reveal that this individual exhibits up to 33% of Ukraine_N and 66% of CHG, supporting high amounts of steppe-related ancestry. When modeled with close chronology populations the individual requires a single source related to the Corded_Ware (Table S5). We tried to assess if the signal could, however, correspond to similar genetic populations but more contemporary and geographically closer to VERT-113, such as Srubnaya, using the statistic *f*_4_(Mbuti, VERT-113; Poland_Southeast_CordedWare, Russia_Srubnaya), but the results (*f*_4_ = 0.00003, Z = 0.11) show that there is no statistical relationship, which indicates no evidence supporting the Srubnaya origin.

Individual VERT-114, dated around the LBA, showed a genetic position close to Bell-Beaker populations in PCA and ADMIXTURE. This individual shows a higher influx of ancestry from WHG than from EHG populations *f*_*4*_(Mbuti, VERT-114; WHG, EHG,) − 0.002, Z score = − 8,64), similar to the results obtained for the aggregate group of 22 Verteba individuals. qpAdm results for this individual show that a single model with a Bell_Beaker population as a single source works. Many of the two-way models involve populations related to Ukraine_Globular_Amphora and to Steppe populations, with about 60% of ancestry from the former and the rest from the latter. We also explored the possible connections between this individual and the Cimmerians, who established themselves in present-day Ukraine around the year 1000 BCE, being contemporary to VERT-114. Both *f*_4_(Mbuti, VERT-114; Moldova_Cimmerian.SG, Poland_Southeast_BellBeaker) *f*_4_ = 0.00428 Zscore = 7.171) and *f*_4_(Mbuti, VERT-114; Moldova_Cimmerian.SG, Hungary_EBA_BellBeaker) *f*_4_ = 0.003301, Zscore = 4.043) showed a clear affinity of VERT-114 to the Bell_Beaker individuals over the Cimmerians. This individual can also be modeled as an admixture between Turkey_N (67%) and Ukraine_N (33%) (Table S6).

We used the approach presented in Ringbauer et al.^48^, to explore the presence of runs of homozygosity (ROH) in the sample. We observed that the samples tested present very few parts of the genome under ROH (Fig. S3), meaning that the individuals were part of large populations. An exception was VERT-100 who showed long ROH segments, suggesting that this individual was an offspring of related individuals.

### Phenotypic positions

We genotyped 105 SNPs linked with metabolic, pigmentation and pathogen resistance phenotypic traits. Pseudohaploid genotypes are shown in Table S10. From these genotypes it is evident that none of the tested individuals from Verteba cave was lactose tolerant as all are homozygous for the non-tolerant variants for SNPs rs4988235^49^ and rs182549^50^. It is also interesting to remark that, except for two individuals, the majority of individuals from Verteba cave have the variant of SNP rs12913832 associated with blue eyes and the other two associated with dark ones^51^.

## Discussion

The CTCC is an important archaeological complex that brought farming to Eastern Europe^16,18^. Prior to our research, the CTCC genomic record consisted of only four individuals from Verteba Cave and four individuals from Moldova, nevertheless, the previously reported diversity within the CTCC^37^ showed that more research on the genetic diversity of this culture is needed to understand its origin, dynamics, and collapse. Recent publications have revealed the utility and the relevance of large-scale projects focused on specific sites^43,52^. Here, we have presented genomic data from 20 individuals buried in Verteba Cave that are dated to the fourth, second and first millennia BCE. The genetic analysis of these individuals has revealed important genetic turnovers both in the Early Bronze Age and during the Late Bronze Age. In the future, more individuals should be sequenced to clarify these observations, in particular to obtain more individuals from the third millennium BCE onward, as the genomic record in Ukraine from the Bronze Age is limited to six individuals from the second and third millennia BCE. Importantly, we also provide eight new radiocarbon dates, which are extremely relevant, as previous studies have demonstrated the presence of diverse material in Verteba Cave’s, caused by repeated use of the site from at least the Mesolithic up until modern times^28^.

Previous analyses of CTCC individuals’ mitochondrial DNA HVRI indicated their close maternal ancestry with early Neolithic groups, with lineages that are representative of the Neolithic ‘package’, including haplogroups H, HV, T, V, J, and K^53,54^. With the exception of two individuals with haplogroup U5a, all the other 18 individuals that were included in our analyses have haplogroups that are similar to Central European Neolithic groups^13^. This diversity is in stark contrast to individuals from earlier non-agricultural Neolithic sites from the Ukraine that have only haplogroup U, likely the result of continuity with previous Mesolithic hunter-gatherers (HG). The mtDNA haplogroup diversity suggests that local populations were largely replaced by those associated with the Trypillian culture. The majority of VC individuals exhibit the G2a2 Y haplogroup, which is widely present in Anatolia-related Neolithic European individuals^5,13^. The other identified haplogroups, C1 and I2, have been also reported among European Neolithic populations, pointing to an origin of the CTCC individuals without a sex-biased migratory past, which contrasts with the steppe migrations during the Bronze Age^55^.

Population genetic analyses indicate that the individuals buried at Verteba Cave during the Late Eneolithic (3790–3535 cal BCE) genetically resemble other previously published CTCC individuals, and are closely related to other published CTCC individuals in Moldova^37^. These observations broadly suggest that Eneolithic CTCC individuals descended from the same, or closely related, population that spread the Neolithic across most of Europe and without little or no sign of admixture with earlier Ukrainian Mesolithic or Neolithic groups composed of hunter-gatherer-related ancestry and specifically pointing towards the Baden individuals from Hungary. In fact, most of the Trypillian individuals can be modeled by Eneolithic populations from Europe that have steppe ancestry, however four out of the 20 individuals could be modeled as Moldovan Trypillians. These results in the qpAdm modeling suggest that there were differences in the ancestry composition of the Trypillians of Verteba Cave, which could be linked to the proportion of HG in the individuals, although this variability is not substantial enough to differentiate the individuals into different populations.

Previous studies of CTCC individuals could not provide a clear origin for the HG component of CTCC-associated groups. Here, despite observing that models including Ukranian_N individuals and WHG seem to work, the *f*_*4*_-statistics suggest that the source of the HG component would be mainly WHG. In addition, not a single qpAdm model using EHG as a source works, which supports that observation. The significant proportion of WHG ancestry found in the Trypillians (up to 18%) might be related to the hunter gatherer resurgence seen in other Middle Neolithic populations of Central Europe, likely due to admixture with groups in the west who already had a higher WHG component derived from Anatolia-related Neolithic groups prior to the origin of the CTCC^8,13,56^. This would also indicate that the HG Neolithic populations from Ukraine did not contribute much ancestry to the Trypillians. In addition, we also observe the presence of steppe-related ancestry in these individuals, as was revealed in Moldova^37^, although the proportion in the Verteba individuals is lower, which could correlate with the age of the individuals suggesting a continuous pulse from the East to the West gradually increasing the Yamnaya-related ancestry during the fourth millenium BCE.

Individual VERT-113, dated to the Middle Bronze Age (1960–1770 cal BCE [2σ]), has an ancestry profile that is quite different from the earlier CTCC individuals. There is significantly more Caucasus HG/Yamnaya and EHG ancestry, and thus this individual was related to the Yamnaya expansions. qpAdm results suggest a link between VERT-113 and Corded Ware populations from Poland, pointing to a similarity between this individual and these populations. Also, this is the only individual with a higher genetic affinity to the Ukraine_N than to the WHG, suggesting that the population that originated MBA in the second millenium BCE may have had shared affinities with the Ukraine_N populations.

Interestingly, VERT-114 (Late Bronze Age) does not show many genetic connections with MBA VERT-113 according to the *f*_*3*_ values, who is clearly associated with the Yamnaya pastoralists. The genomic composition of VERT-114 suggests a relationship with Beaker-related populations, despite being almost 1000 years younger than the end of the Bell Beaker phenomenon^57^, and with a date that would be more coincident with the Cimmerians or Scythians^58^. However, no qpAdm models with these cultures work and the *f*_*4*_ results do seem to confirm the similarity with the Bell Beakers over the Cimmerians. The genetic background of this individual, with its strong western affinities, supports the evidence shown in Narasimhan et al.^59^ of a western influx into the Steppe during the Late Bronze Age. Further sequencing and analysis of individuals from the cave and the area surrounding VC dating from the third millennium BCE will be critical for exploring cave use after abandonment by the CTCC-related peoples.

The results of our paleogenomic analysis have important implications for understanding the Neolithisation process in far eastern Europe. As the populations of the CTCC expanded from Romania and Moldova into the forest-steppe areas of western and central Ukraine, they would have come into contact with populations associated with the indigenous Bug-Dniester culture, a group whose subsistence system focused primarily on foraging^60^. This group was likely descendant from Mesolithic hunter-gatherers. The paleogenomics of the Verteba Cave individuals suggest that local Mesolithic hunter-gatherers did not contribute significantly to later Trypillian ancestry, indicating that the process to Neolithisation in western Ukraine was the product of substantial migration rather than indigenous adoption of agricultural practices.

Our results also provide support to the idea that a long-lasting frontier existed between the sedentary agriculturalists of the forest-steppe ecozone and the neighboring nomadic pastoralists from the Pontic Steppe. This frontier is characterized by drastic contrasts in material culture and subsistence regimes, and was likely maintained in prehistory due to these factors as well as by major linguistic differences^60^. Documenting the lack of admixture on this cultural frontier is key to understanding the context from which the Yamnaya migration occurred^8^.

In conclusion, the results show that Verteba Cave represents a significant mortuary site that connects East and West. The genetic structure of the CTCC peoples includes ancestry related to both earlier hunter-gatherers from the west and farmers from the Near East, and one that is genetically distinct from those of Moldovan CTCC peoples. The lack of local ancestry associated with Ukrainian Neolithic hunter-gatherers suggests that these farmers mostly replaced local foragers and did not mix with the neighbouring steppe populations. Additionally, during the Bronze Age, Verteba Cave was used by successive waves of nomadic pastoralists from the east that eventually brought significant genetic and cultural changes to Europe that eventually mixed with the local descendants of Trypillia-culture population. Additional genomic sampling from these later time periods will help to answer questions of site chronology and possibly indicate how the Trypliian culture eventually collapsed.

## Materials and methods

To perform the present study, 23 samples were collected from Verteba cave (Ukraine). Due to low coverage, two samples were not included in the final analyses. The complete description of the methods can be found in the Supplementary Information section.

### AMS radiocarbon analysis

The ages of individuals from this study were determined using AMS ^14^C dating; here we report eight new dates run at the Pennsylvania State University Accelerator Mass Spectrometry Laboratory (lab code: PSUAMS) and the Oxford Radiocarbon Accelerator Unit (lab code: OxA) (see Table S1).

Bone collagen extraction for ^14^C and stable isotope analysis was extracted and purified at the Pennsylvania State University using a modified Longin method with ultrafiltration^61^. In cases where this method returned an unacceptably low gelatin yield, samples were processed according to the XAD amino acid purification method^62^. Samples were prepared first by manual cleaning adhering sediment and removing exposed surfaces with an X-acto blade. This was followed by demineralization for 24–36 h in 0.5 N HCl at 5 °C. The pseudomorph was then rinsed to neutrality in multiple changes of Nanopure H_2_O, before being gelatinized for 10 h at 60 °C in 0.01 N HCl. The resulting gelatin was lyophilized, visually inspected and then weighed to assess bone collagen preservation. Rehydrated gelatin solution was pipetted into pre-cleaned Centriprep^63^ ultrafilters (retaining 30 kDa molecular weight gelatin) and centrifuged 3 times for 20 min, diluted with Nanopure H_2_O, and centrifuged 3 more times for 20 min to desalt the solution. Carbon and nitrogen concentrations and stable isotope ratios were measured at the Yale Analytical and Stable Isotope Center with a Costech elemental analyzer (ECS 4010) and Thermo DeltaPlus analyzer. Sample quality was evaluated by examining the % crude gelatin yield, %C, %N and C:N ratios before AMS ^14^C dating. C:N ratios for the dated samples fell between 3.15 and 3.42, indicating acceptable collagen preservation^64^. Collagen samples were combusted for three hours at 900 °C in vacuum-sealed quartz tubes with CuO and Ag wires. Sample CO2 was reduced to graphite at 550 °C using H2 and a Fe catalyst, with reaction water drawn off with Mg(ClO_4_)_2_^65^. Graphite samples were pressed into targets in Al cathodes and loaded on the target wheel for AMS analysis. The ^14^C ages were corrected for mass-dependent fractionation with measured δ^13^C values^66^ and compared with samples of Pleistocene whale bone (backgrounds, 48,000 ^14^C BP), late Holocene bison bone (~ 1850 ^14^C BP), late AD 1800s cow bone and OX-2 oxalic acid standards for calibration. Sample preparation protocols at OxA follow similar standards and have been published in depth elsewhere (Brock et al. 2010).

Dates were calibrated according to the IntCal20 Northern Hemisphere calibration curve^67^ using OxCal 4.4^68^. Six of the eight dates display a tight distribution ranging from 3790 to 3535 cal BCE (2σ), with mean results spread across a single ~ 80-year period from 3720–3640 cal BCE. These results are comparable with dates from other sites in Romania, Moldova and Ukraine dating to the latter part of period Trypillia CI, and are consistent with the late Trypillia CI (Shypynetska local group) occupation identified at the site (Nikitin et al. 2010). A single date corresponds with the Middle Bronze Age (PSUAMS-3153; VERT-113), dating to the range of 1960–1770 cal BCE (2σ).

### DNA extraction and library preparation

All laboratory work was performed in dedicated ancient DNA laboratories at University College, Dublin. These facilities are physically located from other molecular biology laboratories, and measures are taken to minimize contamination of ancient individuals, including head-to-toe suits, face masks, hair nets, multiple layers of gloves, bleaching of all surfaces and UV decontamination of all (non-sensitive) reagents. All laboratory tools used to process samples were decontaminated using bleach (1:5 concentration) and UV irradiated in a cross-linker. The final step of library preparation (amplification) was performed outside the ancient DNA laboratory. We included extraction negative controls (no powder), library and PCR negative controls (extract was supplemented by water) in every batch of samples processed and carried them through the entire wet laboratory processing to test for reagent contamination.

Samples were initially UV irradiated on both sides for ~ 10 min. We targeted the inner ear region of the petrous bone^69,70^ using a sandblaster (Renfert). Fragments of the cochlea were then powdered using a mixer mill (Retsch Mixer Mill 400). Twenty-three petrous bone samples were initially screened. Using ~ 75 mg of powder, DNA was extracted using an optimized DNA extraction protocol^71^. Illumina sequencing libraries were constructed using 12.5-25uL of extract, amplified using Accuprime Pfx Supermix (Thermo Fisher Scientific), following Gamba et al.^72^; a protocol adopted from^73^. Quality assessment of the amplified library was performed on an Agilent 2100 Bioanalyzer and a Qubit 2.0 Fluorometer. All amplified libraries were initially screened using an Illumina MiSeq. After screening, additional libraries were sequenced to ~ 1X on the NextSeq platform.

### Bioinformatic analysis

Sequencing reads were trimmed using cutadapt (Version. 1.2.1)^74^ and aligned to the human reference genome (GRCh37), with the mitochondrial genome replaced by the revised Cambridge reference sequence (rCRS) using BWA^75^ (Version 0.7.5). Duplicate mapped reads were removed using Picard Tools^76^. Reads with mapping qualities below 30 were also removed. Unique and filtered reads were analyzed with qualimap-2^77^ to assess the coverage of the genomes. MapDamage-2^78^ was used to estimate the level of deamination and the authenticity of the data. We have clipped two bases per read end to minimize the effect of damage.

Pseudo-haplotypes were called using sequenceTools^79^ filtering the calls with mapping and base quality below 30. As a reference panel we used the positions of the 1240 k capture dataset^8^. The Verteba calls were merged with a panel of 750 modern individuals from 46 populations^3^ and 611 individuals from 67 ancient populations^3–5,8,13,37,45,56,57,80–89^. Molecular sex was determined using ry_compute.py^90^, we also explored the presence of familiar relationships with READ^91^.

Mitochondria aligned reads were processed with Schmutzi^92^ to generate a consensus sequence of the mitochondrial genomes using –uselength option. We determined the mitochondrial haplogroups of the mitochondrial consensus sequences with Haplogrep v2.0^93^. Y chromosome haplogroups of male individuals were determined using Yleaf^94^.

Principal component analysis was built with 597,573 SNPs and 750 modern genomes using smartpca from Eigensoft package^46^. Resulting data was plotted using R^95^. Ancient samples were projected in the PCA built with the modern ones using the option lsqproject. Two rounds of outlier removal were used. Results were plotted with R.

An unsupervised ADMIXTURE analysis was performed with ADMIXTURE^47^. 611 ancient individuals, 2068 modern individuals together with the 20 Verteba individuals were used for the analysis. From the 597,573 SNPs of the Human Origins dataset was filtered removing SNPs with MAF below 0.05 and more than 5% of missing sites. Filtered SNPs were pruned by linkage-disequilibrium (LD) using PLINK 1.9^96^ flag –indep-pairwise with a windows size of 200 SNPs, advanced by 50 SNPs and establishing an r2 threshold of 0.4. The ADMIXTURE analysis was performed with 417,913 SNP s, with K ranging from 2 to 15 and 10 bootstrap replications. Admixture was plotted with R.

*F*-statistics were run using admixtools^46^ in the form *f*_3_(Test, X; Mbuti) using all the ancient European populations available. D statistics were also run using the same package. We have used the form *f*_4_(Mbuti, Test; PopA, PopB) using a list of possible sources of Hunter-Gatherer, Neolithic and steppe components. In this analysis, we excluded results with less than 100,000 shared SNPs.

We performed qpAdm using the admixtools package^46^ In this analysis we have used the same proxies as the ones used for D statistic plus Verteba in case of modeling VERT-113 and VERT-114 individuals and setting allSNPs: NO. The list of samples included in each category is displayed in table S6. As outgroups we used: Russia_Kostenki14.SG, Italy_North_Villabruna_HG,Han.DG,Mbuti.DG,ONG.SG,Papuan.SDG,Russia_MA1_HG.SG,Ust_Ishim. In a second round we added the rest of tested populations to the right making the models compete between them as described in Harney et al.^97^. We also performed qpWave analysis to assess the presence of substructure in the Verteba_Trypillia population using the same software and right population set listed below, the threshold was set to a p-value of 0,05.

We calculated the ROH segment distribution following the protocol described in Ringbauer et al.^48^. The phenotypic positions analyzed were genotyped using the pseudo haploid calls. The frequencies of the present day populations were obtained from the 100 genomes data^98^.

## Supplementary Information

Supplementary Information 1.

Supplementary Information 2.

## Data Availability

Sequencing reads have been deposited in the European Nucleotide Archive (ENA) with the accession code PRJEB38797.
