# Supplementary material for: Genomes from Verteba cave suggest diversity within the Trypillians in Ukraine
Source: Sci Rep. 2022 May 4;12:7242. doi: 10.1038/s41598-022-11117-8 (PMC9068698; doi:10.1038/s41598-022-11117-8)
Supplement: Supplementary file 1 — Supplementary Information 1. [file 41598_2022_11117_MOESM1_ESM.docx]

**Genomes from Verteba cave suggest diversity within the Trypillians in Ukraine**

Pere Gelabert , Ryan W. Schmidt , Daniel M. Fernandes, Jordan K. Karsten, Thomas K. Harper, Gwyn D. Madden , Sarah H. Ledogar, Mykhailo Sokhatsky, Hiroki Oota, Douglas J. Kennett, and Ron Pinhasi

Contents

S1: Archaeological Context of Verteba Cave and Sample

S2: Supplementary methods

S3: Supplementary Figures

S4: References

S1: Archaeological Context of Verteba Cave and Sample Information

Despite the fact that western Ukraine is home to a large number of Trypillia-culture settlements, very few burials have been located, limiting our knowledge of mortuary behavior and precluding previous bioanthropological study of this group. Burials and cemeteries are rare in and around Trypillia sites and from all phases (Table S11) . A few isolated and poorly preserved burials have been found under the floors of houses dating to the early period of the CTCC [^1^](https://paperpile.com/c/Xt4w48/E5a1I). This practice, while rare among the sites of the Trypillia culture, is a characteristic of Early and Middle Neolithic mortuary behavior observed across southeastern Europe [^2^](https://paperpile.com/c/Xt4w48/pKIfS). Several explanations for the general absence of Trypillia burials have been proposed, including cremation, or open-air excarnation and exposure[^3^](https://paperpile.com/c/Xt4w48/F80Zt). Alternatively, these populations may have buried their dead in locations far from settlement sites [^4^](https://paperpile.com/c/Xt4w48/zlFY7).

The use of cemeteries, a practice typical of Mesolithic and Neolithic Ukrainian sites, has only been found at Late Trypillia sites. Chapaievka (~3,700–3,400 BCE), the earliest Tripolye cemetery, is located next to a settlement just south of Kyiv[^3^](https://paperpile.com/c/Xt4w48/F80Zt). It is situated close to the steppe frontier; kitchenware at Chapaievka is very similar to the neighboring Sredni Stog (pre-Yamnaya) ceramics, indicating a close relationship between this steppe culture and the Eastern Trypillia [^4^](https://paperpile.com/c/Xt4w48/zlFY7). This cemetery contains 31 extended burials of men, women, and one child oriented to the west and associated grave goods including ceramics, lithic tools, and a female figurine [^4^](https://paperpile.com/c/Xt4w48/zlFY7). A larger cemetery in Moldova, Vykhvatyntsi, has yielded more than 70 burials [^5^](https://paperpile.com/c/Xt4w48/9P2DI). Individuals were buried in flexed positions in burial pits encased with stones [^4^](https://paperpile.com/c/Xt4w48/zlFY7). Like Chapaievka, Vykhvitnitsi is also located near the steppe frontier, and it is possible that in both cases Trypillia populations (over 300 km distant from one another) were gradually integrating the steppe custom of inhumation burial into their own mortuary practices. Late Trypillia cemeteries of the Sofiyivska local group of the Eastern Trypillia culture (~3,300–2950 BCE) include both inhumation and cremation burials [^6^](https://paperpile.com/c/Xt4w48/0eN4)..

Verteba Cave is the only known large accumulation of human skeletal remains from a cave or rock shelter from any period of the Trypillia culture. The cave is located on a terrace approximately 100m from the Siret River and 1.5 km from the village of Bilche Zołote in a geographic region known as the Podilian Plateau. It is one of many caves in western Ukraine and is part of an expansive Miocene gypsum karst that spreads over 20,000 km^2^ in the region [^7^](https://paperpile.com/c/Xt4w48/0fjal). The Verteba Cave karst system entails an extensive network of corridors and caverns totaling around 8 km in length. There are no known freshwater sources in the cave. The modern entrance to the subterranean cave is a depression in the ground, allowing people and animals to enter the cave easily on foot.

Verteba Cave was uncovered as an archaeological site in the 1820s [^8^](https://paperpile.com/c/Xt4w48/TRl2). Surveys of the cave have identified multiple areas of human activity based on findings of ceramics, lithic tools, bones, and charcoal. Initial excavations were carried out in the 1870s, 1890s, and early 1900s[^8–12^](https://paperpile.com/c/Xt4w48/TRl2+wc3s+q6id+3viN+gZto) Modern excavations at Verteba Cave started in 1996 and continue to the present day under the direction of co-author M. Sokohatskyi (Borshchiv Regional Museum of the Ukrainian Ministry of Culture and Arts). This work has demonstrated that surface strata have been disturbed by prior excavation and intrusions throughout history; however, deposits against the cave walls and in sites that are difficult to access, as well as lower strata, have remained mostly untouched by human activity. Bioturbation appears to have affected most sites to a small degree. Water dripping in from the surface, and animals dwelling in the cave (e.g., rodents, bats, and badgers) have all had minor effects on the spatial organization of artifacts and bones in the cave.

Excursions into the cave by archaeologists and speleologists have uncovered over 20 archaeological sites associated with Trypillia culture. These sites have been identified through surveys, and confirmed through test pits. All sites have Trypillia ceramics, indicating their connection with the Trypillia occupation of Verteba Cave. Most ceramic evidence is consistent with utilization of the cave during the later phases of the Eneolithic, although there are two human bones that have been previously dated to the Bronze Age [^13^](https://paperpile.com/c/Xt4w48/kXhZj). Analysis of the human burials in the context of associated faunal remains suggests the cave was used for ritual functions that combined mortuary ceremonies and feasting [^14^](https://paperpile.com/c/Xt4w48/j2FYq). Furthermore, bioarchaeological analyses of human skeletal remains from Verteba Cave has allowed for a reconstruction of the Trypillia diet as being dominated by cereals and a determination that the Trypillia population of Verteba Cave experienced considerable amounts of violent trauma [^15–17^](https://paperpile.com/c/Xt4w48/diFcp+ijirQ+LTvjO).

Table S11: Chronology of the Trypillia culture in Ukraine, from Rassamakin and Menotti (2011) [^18^](https://paperpile.com/c/Xt4w48/H9Kd).

| Phase | Date |
| --- | --- |
| A | 5100/5000–4700/4600 cal BC |
| BI | 4700/4600–4400/4300 cal BC |
| BI/II | 4400/4300–4200/4100 cal BC |
| BII | 4200/4100–3900 cal BC |
| CI | 3900–3450/3350 cal BC |
| CII | 3450/3300–3000/2900 cal BC |

S2: Supplementary methods

**Sampling and DNA Extraction**

All human skeletal samples selected for genomic sequencing, came from an intact cultural layer buried below a layer of fill soil. At each of the sites, the samples were excavated from archaeological contexts with artifacts recognized as being diagnostic of Trypillian material culture, including ceramics, anthropomorphic figurines, and zoomorphic figurines. In addition to these artifacts, the human remains were found in association with numerous non-human faunal skeletal elements, chipped-stone sickle blades, and ground stone mortars and pestles. The faunal elements were primarily from domesticated animals, including cow, sheep, goat, dog, and pig[^14^](https://paperpile.com/c/Xt4w48/j2FYq) [^14^](https://paperpile.com/c/Xt4w48/j2FYq). Beyond having diagnostically Trypillian artifacts, the archaeological contexts of the Verteba Cave sites studied here are consistent with Eneolithic assemblages previously attributed to the Trypillians ^[4](https://paperpile.com/c/Xt4w48/zlFY7)^. There is no Bronze Age material found in Verteba cave, which suggests that the presence of these individuals is sporadic[^14^](https://paperpile.com/c/Xt4w48/j2FYq).

We sampled the petrous portion of the human temporal bone. Specifically, we targeted the osseous inner canal region. Previous studies have shown that this region contains higher amounts of endogenous DNA compared to other regions within the petrous. Samples were collected in two ways. Lab IDs VERT008, VERT-015, and VERT-035 were collected on-site at Verteba Cave. Sterile collection conditions were utilized, which consisted of wearing coveralls, double gloves, and masks to limit modern sources of contamination. All other bones used in this study were sampled from the collection at the University of Wisconsin-Oshkosh by cutting out the petrous portion using a dremel tool. In both cases, samples were placed into sterile bags and brought to the lab at University College, Dublin for further processing.

All bone samples were UV-irradiated in a cross-linker for 15 minutes on each side. A sandblaster (Renfert) was then used to isolate the cochlea from the petrous pyramid. A MixerMill (Rensch) was then used to powder the sample, resulting in 50-75 mg of bone powder for extraction. All samples were then extracted according to the silica-based protocol of Dabney et al. with the modification of the binding apparatus using Qiagen MinElute columns being replaced with the Roche Large Volume Viral Extender tubes. For each sample, 50–75 mg of powder was digested in 1mL of extraction buffer containing 0.45 M EDTA (pH 8.0) and 0.25 mg/mL of proteinase K. After around 18h incubation, samples were centrifuged at 13,000 rpm for 2 m. The supernatant was then added to 13 mL of binding buffer consisting of 5M Guanidine hydrochloride (MW 95.53), 40% Isopropanol, 0.05% Tween-20, and 9 mM Sodium Acetate in the Roche Extender columns. The 50-mL columns were centrifuged for 4 m at 1,500 x g. The columns were then washed twice in 650 mL of PE buffer (Qiagen), centrifuging at 6,000 rpm between washes. After the second wash, samples were centrifuged at 13,000 rpm. Samples were eluted two times in 25 µL of EBT after 10 m incubation at 37°C and centrifuged at 13,000 rpm for 30 sec, for a total of 50 µL extract. Extraction batches included negative controls (no DNA template).

**Library construction and sequencing**

Illumina blunt-end libraries were built following the protocol of Meyer and Kircher, with modifications by Gamba et al [^19^](https://paperpile.com/c/Xt4w48/enFH). Libraries were prepared using 12.5-25 µL of DNA extract in a 70µL reaction volume for the blunt end repair step and 40µL reaction volumes for the adapter ligation and adapter fill-in steps. Blunt end repair was performed using NEB End Repair module. Samples were incubated for 15 m at 25°C followed by 5 m at 12°C. Ligation was performed using T4 DNA ligase buffer (10X), PEG-4000 (50%), adapter mix following Meyer and Kircher, and T4 DNA ligase (5U/µL). Samples were incubated for 30 m at 22°C. Adapter fill-in was performed using Thermopol Reaction Buffer (10X) dNTPs (10mM each), and Bst large fragment (8U/µL). Samples were incubated for 30 m at 37°C and enzyme inactivation was completed by incubating for 20 m at 80°C. Sample cleanup between blunt end repair and ligation, and between ligation an adapter fill-in using the MinElute PCR Purification kit (Qiagen) according to manufacturer’s instructions. Every batch of libraries contained a negative control using water.

The library amplification step was set up using Accuprime pfx Supermix, primer IS4 (10µM), a specific indexing primer (0.2µM), and 3µL of library template, for a total of 25µL reaction mixture. Amplification took place under the following thermal cycling conditions: 5 min at 95°C; 12 cycles of 15 sec at 95°C, 30 sec at 60°C and 30 sec at 68°C; and a final extension step of 5 min at 68°C. The resulting PCR product was purified using the MinElute PCR Purification kit (Qiagen) as described previously. Quantification and quality assessment of the amplified libraries was performed on an Agilent 2100 Bioanalyzer, using a DNA-1000 chip, and a Qubit 2.0 Fluorometer, following manufacturer's protocols.

One amplified library from each sample was first screened on an Illumina MiSeq platform (Core Genomics Center, University College, Dublin) using 70 bp single-end sequencing and following the manufacturer’s instructions for multiplex sequencing. The results of this sequencing were used to estimate endogenous DNA contents. After screening, an additional 19 libraries were single-end sequenced on an Illumina NextSeq using the High Output 550/500 kit, following manufacturer’s instructions for multiplex sequencing.

**Data Authenticity and contamination estimation**

To authenticate the data we examined the deamination pattern with mapDamage 2.0 [^20^](https://paperpile.com/c/Xt4w48/jlZl) . The amount of deamination in the read ends is shown in Table S12. All samples exhibit deamination values concordant with the age of the samples.

Supplementary Table S12: Deamination rate of the sequenced samples

| Sample | 5’ C>T (%) | 3’ G>A (%) |
| --- | --- | --- |
| VERT-113 | 9,3% | 9,6% |
| VERT-107 | 13,4% | 11,72% |
| VERT-105 | 12,4% | 10,5% |
| VERT-104 | 10,8% | 9,4% |
| VERT-103 | 13,3% | 13% |
| VERT-100 | 14,6% | 12,4% |
| VERT-015 | 10,3% | 8,4% |
| VERT-008 | 10,5% | 10,8% |
| VERT-028 | 11,7% | 11,5% |
| VERT-029 | 11,8% | 9,3% |
| VERT-030 | 13,25 | 12,3% |
| VERT-031 | 9,6% | 9,3% |
| VERT-033 | 11,4% | 10,9% |
| VERT-035 | 9,2% | 10,2% |
| VERT-111 | 19,1% | 18,5% |
| VERT-114 | 23,6% | 17% |
| VERT-115 | 23,1% | 22,6% |
| VERT-117 | 21,1% | 21,2% |
| VERT-118 | 19,0% | 19,8% |
| VERT-106 | 25,9% | 26% |

We estimated the contamination of the samples using two methods: Mitochondrial contamination estimation with Schmutzi[^21^](https://paperpile.com/c/Xt4w48/HC8pA) and X chromosome contamination in males with ANGSD[^22^](https://paperpile.com/c/Xt4w48/arVYa), both using default values. In both cases we did not detect a significant amount of contamination in any of the sequenced libraries (Table S2).

S3: Supplementary figures

Figure S1: Admixture plots using K1-20 with 100 bootstrap replications.


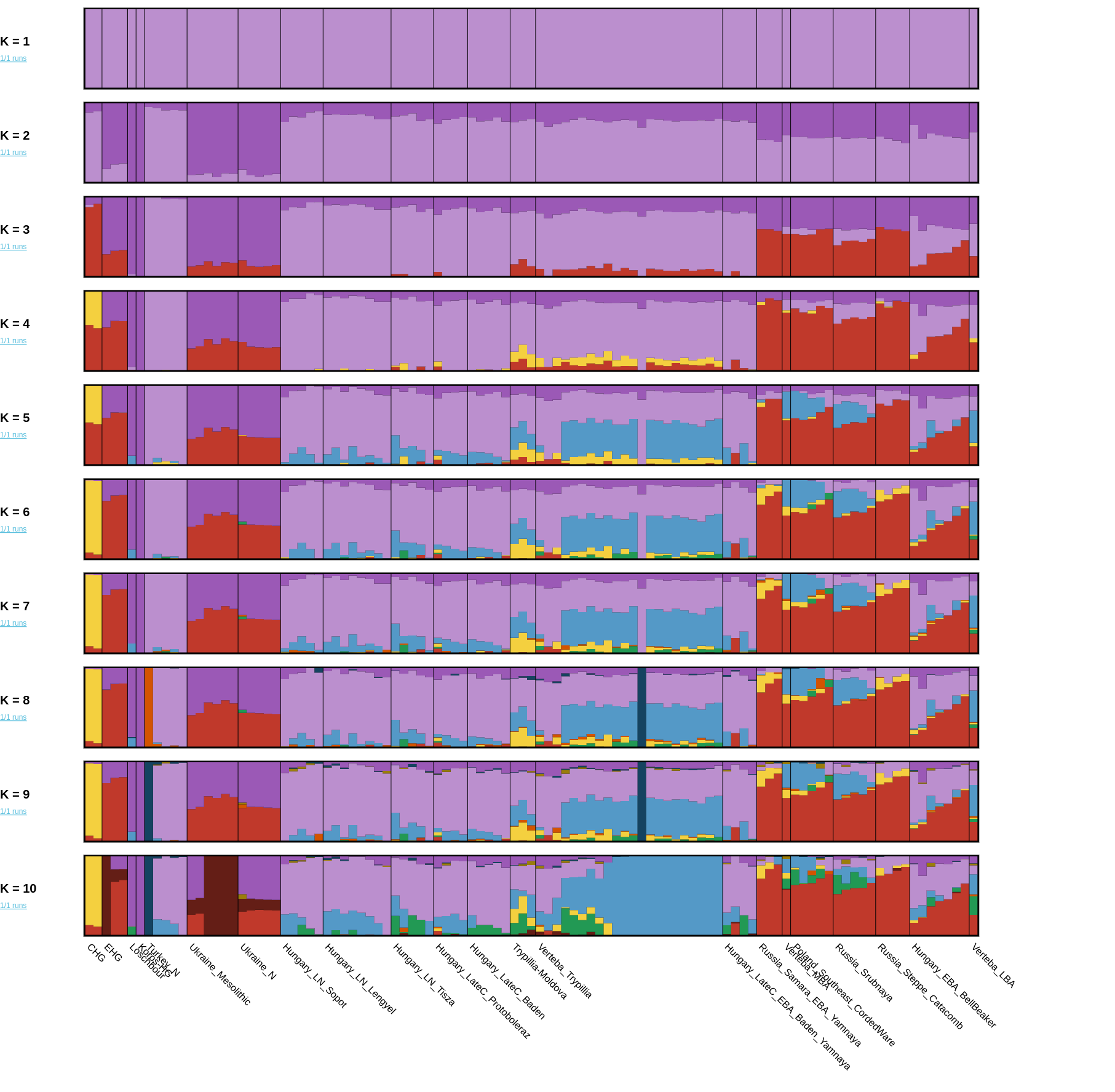


Figure S2: D-statistics results of the tested samples in the combination *f*_4_(Mbuti,Verteba;Russia_Samara_EBA_Yamnaya,X).


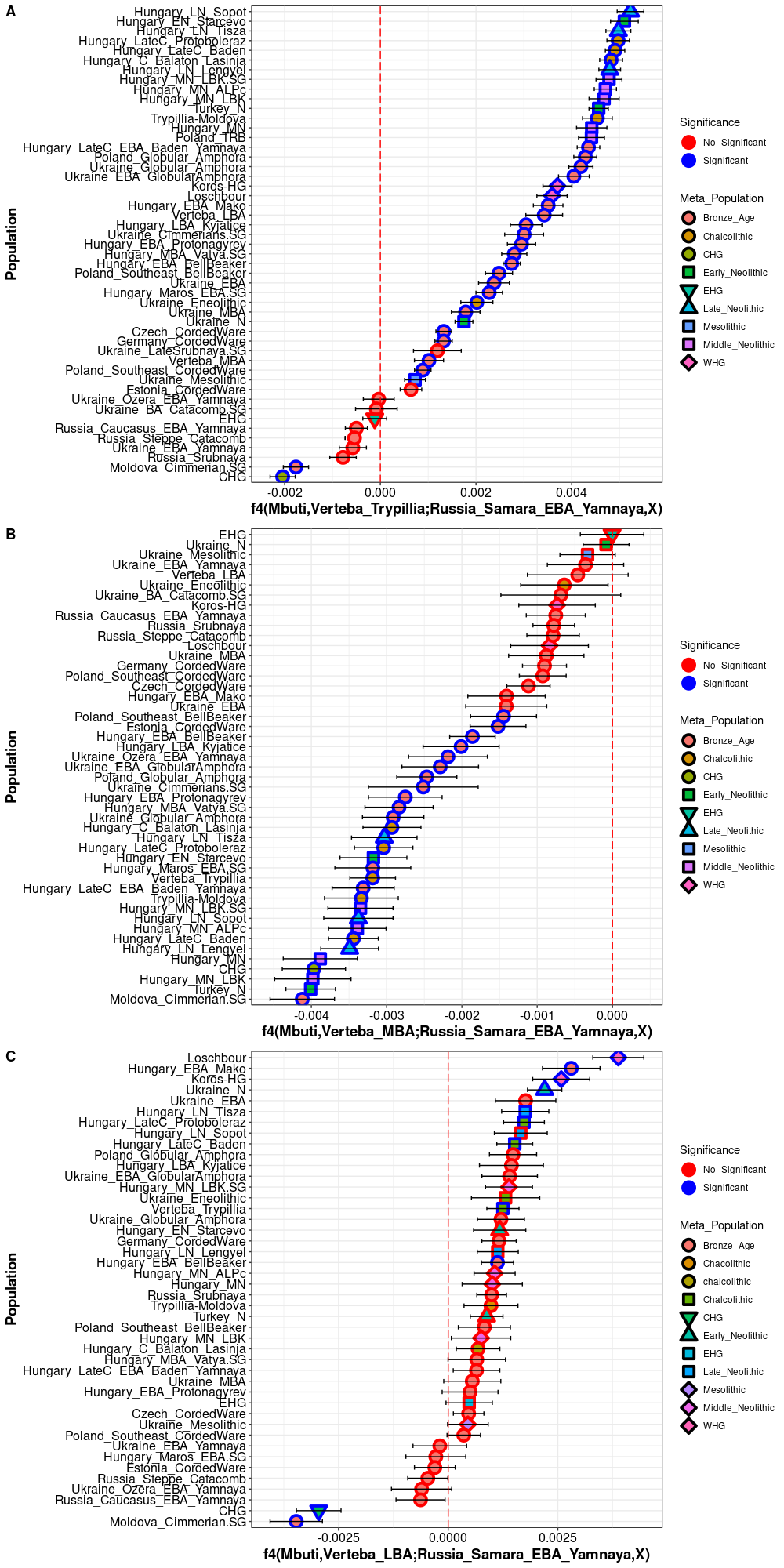


Figure S3: sum of ROH of the studied genomes using the method described in Ringbauer et al 2021. [^23^](https://paperpile.com/c/Xt4w48/7nb6)


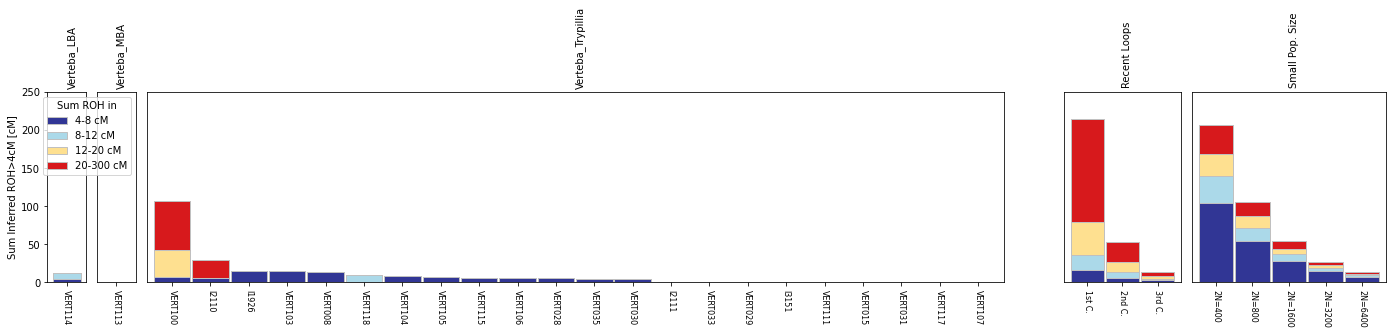


S4: References

1. [Movsha, T. G. The Tripolye-cucuteni and the Lengyel-Polgar cultures. *Balt.-Pontic Stud.* (2000). at <](http://paperpile.com/b/Xt4w48/E5a1I)<https://repozytorium.amu.edu.pl/handle/10593/13171>[>](http://paperpile.com/b/Xt4w48/E5a1I)

2. [Kadrow, S. Gender-differentiated burial rites in Europe of the 5th and 4th millennia BC: attempts at traditional archaeological interpretation. (2008). at <](http://paperpile.com/b/Xt4w48/pKIfS)<https://repozytorium.ur.edu.pl/bitstream/handle/item/2866/2%20kadrow-gender.pdf?sequence=1&isAllowed=y>[>](http://paperpile.com/b/Xt4w48/pKIfS)

3. [Anthony, D. W. *The Horse, the Wheel, and Language: How Bronze-Age Riders from the Eurasian Steppes Shaped the Modern World*. (Princeton University Press, 2007).](http://paperpile.com/b/Xt4w48/F80Zt)

4. [Zbenovich, V. G. The Tripolye culture: Centenary of research. *Journal of World Prehistory* **10,** 199–241 (1996).](http://paperpile.com/b/Xt4w48/zlFY7)

5. [Dergachev, V. A. *Vykhvatinskiĭ*. (Shtiint︠s︡a, 1978).](http://paperpile.com/b/Xt4w48/9P2DI)

6. [Kruts, V. A. *Pozdnetripolskie pamiatniki Srednego Podneprovia*. (Naukova Dumka, 1977).](http://paperpile.com/b/Xt4w48/0eN4)

7. [Klimchouk, A. Gypsum karst in the Western Ukraine. *Int. J. Speleol.* **25,** 20 (1996).](http://paperpile.com/b/Xt4w48/0fjal)

8. [Kadrow, S. & Pokutta, D. A. The Verteba cave: a subterranean sanctuary of the Cucuteni-Trypillia culture in Western Ukraine. *Journal of Neolithic Archaeology* (2016). at <](http://paperpile.com/b/Xt4w48/TRl2)<http://www.diva-portal.org/smash/record.jsf?pid=diva2:1261251>[>](http://paperpile.com/b/Xt4w48/TRl2)

9. [Kirkor, H. Sprawozdanie i wykaz zabytków złożonych w Akademii Umiejętności z wycieczki archeologiczno-antropologicznej w roku 1878. *Zbiór Wiadomości do Antropologii Krajowej* **3,** 12–45 (1879).](http://paperpile.com/b/Xt4w48/wc3s)

10. [Ossowski, G. Sprawozdanie czwarte z wycieczki paleoetnologicznej po Galicji. *Zbiór wiadomości do antropologii krajowej* **18,** 1–28 (1895).](http://paperpile.com/b/Xt4w48/q6id)

11. [Ossowski, G. Sprawozdanie trzecie z wycieczki paleoetnologicznej po Galicji. *Zbiór wiadomości do antropologii krajowej* **16,** 63–95 (1892).](http://paperpile.com/b/Xt4w48/3viN)

12. [Ossowski, G. Sprawozdanie drugie z wycieczki paleontologicznej po Galicyi w roku 1890. (1891). at <](http://paperpile.com/b/Xt4w48/gZto)<https://rcin.org.pl/iae/dlibra/publication/edition/5624>[>](http://paperpile.com/b/Xt4w48/gZto)

13. [Nikitin, A. G. Bioarchaeological analysis of Bronze Age human remains from the Podillya region of Ukraine. *Interdisciplinaria Archaeologica: Natural Sciences in Archaeology* **2,** 9–14 (2011).](http://paperpile.com/b/Xt4w48/kXhZj)

14. [Ledogar, S. H. *A zooarchaeological and geochemical analysis of the faunal remains from the Tripolye site Verteba Cave, Ukraine*. (State University of New York at Albany, 2017).](http://paperpile.com/b/Xt4w48/j2FYq)

15. [Madden, G., Karsten, J. & Heins, S. Cranial Clusters: Mortuary Patterns in Eneolithic Verteba Cave, Western Ukraine. (2015). at <](http://paperpile.com/b/Xt4w48/diFcp)<https://scholarworks.gvsu.edu/fsdg/514/>[>](http://paperpile.com/b/Xt4w48/diFcp)

16. [Karsten, J. K., Heins, S. E., Madden, G. D. & Sokhatskyi, M. P. Dental Health and the Transition to Agriculture in Prehistoric Ukraine: A Study of Dental Caries. *European Journal of Archaeology* **18,** 562–579 (2015).](http://paperpile.com/b/Xt4w48/ijirQ)

17. [Madden, G. D., Karsten, J. K., Ledogar, S. H., Schmidt, R. & Sokhatsky, M. P. Violence at Verteba Cave, Ukraine: New insights into the Late Neolithic intergroup conflict. *Int. J. Osteoarchaeol.* **28,** 44–53 (2018).](http://paperpile.com/b/Xt4w48/LTvjO)

18. [Rassamakin, Y. & Menotti, F. Chronological Development of the Tripolye Culture Giant-Settlement of Talianki (Ukraine): 14C Dating vs. Pottery Typology. *Radiocarbon* **53,** 645–657 (2011).](http://paperpile.com/b/Xt4w48/H9Kd)

19. [Gamba, C., Jones, E. R., Teasdale, M. D., McLaughlin, R. L., Gonzalez-Fortes, G., Mattiangeli, V., Domboróczki, L., Kővári, I., Pap, I., Anders, A., Whittle, A., Dani, J., Raczky, P., Higham, T. F. G., Hofreiter, M., Bradley, D. G. & Pinhasi, R. Genome flux and stasis in a five millennium transect of European prehistory. *Nat. Commun.* **5,** 5257 (2014).](http://paperpile.com/b/Xt4w48/enFH)

20. [Jónsson, H., Ginolhac, A., Schubert, M., Johnson, P. L. F. & Orlando, L. mapDamage2.0: fast approximate Bayesian estimates of ancient DNA damage parameters. *Bioinformatics* **29,** 1682–1684 (2013).](http://paperpile.com/b/Xt4w48/jlZl)

21. [Renaud, G., Slon, V., Duggan, A. T. & Kelso, J. Schmutzi: estimation of contamination and endogenous mitochondrial consensus calling for ancient DNA. *Genome Biol.* **16,** 224 (2015).](http://paperpile.com/b/Xt4w48/HC8pA)

22. [Korneliussen, T. S., Albrechtsen, A. & Nielsen, R. ANGSD: Analysis of Next Generation Sequencing Data. *BMC Bioinformatics* **15,** 356 (2014).](http://paperpile.com/b/Xt4w48/arVYa)

23. [Ringbauer, H., Novembre, J. & Steinrücken, M. Parental relatedness through time revealed by runs of homozygosity in ancient DNA. *Nat. Commun.* **12,** 5425 (2021).](http://paperpile.com/b/Xt4w48/7nb6)
